# Manual Removal of the Placenta after Vaginal Delivery: An Unsolved Problem in Obstetrics

**DOI:** 10.1155/2014/274651

**Published:** 2014-04-09

**Authors:** Fiona Urner, Roland Zimmermann, Alexander Krafft

**Affiliations:** Division of Obstetrics, Department of Obstetrics and Gynecology, University Hospital Zurich, 8091 Zurich, Switzerland

## Abstract

The third stage of labor is associated with considerable maternal morbidity and mortality. The major complication is postpartum hemorrhage (PPH), which is the leading cause of maternal morbidity and mortality worldwide. Whereas in the event of PPH due to atony of the uterus there exist numerous treatment guidelines; for the management of retained placenta the general consensus is more difficult to establish. Active management of the third stage of labour is generally accepted as standard of care as already its duration is contributing to the risk of PPH. Despite scant evidence it is commonly advised that if the placenta has not been expelled 30 minutes after delivery, manual removal of the placenta should be carried out under anaesthesia. Pathologic adhesion of the placenta in the low risk situation usually is diagnosed at the time of delivery; therefore a pre- or intrapartum screening opportunity for placenta accreta would be desirable. But diagnosis of abnormalities of placentation other than placenta previa remains a challenge. Nevertheless the use of ultrasound and doppler sonography might be helpful in the third stage of labor. An improvement might be the implementation of standardized operating procedures for retained placenta which could contribute to a reduction of maternal morbidity.

## 1. Introduction

The third stage of labor is still associated with considerable maternal morbidity and mortality. The major complication is postpartum hemorrhage (PPH), which affects about 5% of deliveries [1, 2]. Therefore it is the leading cause of maternal morbidity and mortality worldwide [3]. In western countries, such as the United Kingdom, it is the fifth most common reason for maternal death after thromboembolism, preeclampsia/eclampsia, genital tract sepsis, and amniotic fluid embolism. It has a mortality rate of 0.39 : 100,000 [4].

Some ten years ago, an editorial titled “The retained placenta—new insights into an old problem” was raising hopes that this problem is to be solved soon [5]. Unfortunately, it is still not.

Whereas in the event of PPH due to atony of the uterus there exist numerous guidelines, recommendations, and flowcharts for its management; in the treatment of retained placenta the general consensus is more difficult to establish. Retained placenta is an important cause of PPH and has an incidence of 1 : 100 to 1 : 300 births [6, 7]. With this paper our aim was to attract the obstetricians' attention to the potential risk of retained placenta in the low risk setting where it occurs without prior warning and to present a possible flowchart for the timing of treatment to reduce blood loss and therefore maternal morbidity.

## 2. The Time Factor

In general it can be said that already the duration of the third stage of labour is contributing to the risk of PPH as the risk of major bleeding is believed to increase with time elapsed after birth. Hence, active management of third stage of labour using prophylactic oxytocics is accepted as standard of care. Active management of the third stage of labour involves administration of intravenous oxytocin, early cord clamping, transabdominal manual massage of the uterus, and controlled traction of the umbilical cord. Should this appear insufficient, the next step is usually manual removal of the placenta (MROP). However, the timing of this manoeuver is difficult as the risk of PPH from leaving the placenta* in situ* has to be weighed against the knowledge that manual removal can itself cause hemorrhage. It should also be borne in mind that the placenta may be delivered spontaneously up to 30 minutes or more after delivery of the child, without major additional blood loss. The management questions that thus need answering are When and how to detect increased blood loss? When to call in support staff? When to contact the anesthesiologist? Observation of routine practice shows that MROP is regularly deferred beyond the limits recommended. In the absence of immediate evidence of increased vaginal bleeding, management is often conservative and expectant, open to several different options, and paying little attention to the time elapsed since birth.

In a study of over 12,000 births, Combs and Laros found that the risk of hemorrhage increased after 30 minutes of placental retention [8]. Similarly, Magann et al. found that the risk of hemorrhage increased with time. In their study, the risk of PPH was already significantly increased at 10 minutes and, using a receiving operator characteristic (ROC) curve, they demonstrated that the optimal cut-off time for the prediction of PPH was 18 minutes, with a sensitivity of 31% and a specificity of 90% [9]. However, delaying the manual removal will lead to the spontaneous delivery of many placentas.

Despite scant evidence it is commonly advised that if the placenta has not been expelled 30 minutes after delivery despite active management, MROP should be carried out under anaesthesia. Clearly, in the published recommendations the choice of timing for manual removal depends on the facilities available and the local risks associated with both PPH and MROP. Thus the 2007 intrapartum guidelines produced for the UK government agency the National Institute for Health and Clinical Excellence (NICE) suggest 30 minutes, [10] whereas the WHO manual for childbirth suggests 60 minutes [11]. Accordingly, a survey in Europe showed that time until manual removal of placenta in the absence of bleeding varies widely between different countries, from under 30 minutes (Spain and Hungary) to 60 minutes and more (The Netherlands) [12].

## 3. Difficulties with Definition 

There are different reasons for retained placenta and there is a wide variety in the nomenclature for disturbances in placental disruption. We believe the following classification is sound: placenta adherens is caused by failed contraction of the retroplacental myometrium, incarcerated placenta is caused by a closed or closing cervix, and placenta accreta is caused by abnormal placental implantation [13]. A part of the placenta or the entire placenta is abnormally adherent to the uterine wall without underlying decidua basalis. In placenta increta the placental villi invade into the myometrium, while percreta placenta is classified as placental villi penetrating through the uterine serosa or the adjacent organs, usually the bladder [14, 15]. As there is a fair probability for detecting cases with percreta placenta before the onset of labour due to ultrasound and/or magnetic resonance imaging and therefore operative delivery can be planned with all necessary precautions, it is almost impossible for the clinician to detect or even distinguish between placenta accreta and increta despite numerous attempts to do so with several imaging techniques.

## 4. Risk Factors

Weeks observed a considerable variation in the retained placenta rate between countries [7]. In less developed countries it is less common (about 0.1% of all deliveries) but has a high fatality rate. In more developed countries it affects about 3% of all vaginal deliveries but is very rarely associated with maternal death. It is suggested that interventions common in the most developed countries such as abortions, uterine intervention, labour induction, and use of oxytocin could be contributing to the increase in retained placenta rate with increasing development.

Commonly named risk factors for disturbances in placental disruption, such as placenta accreta, are history of retained placenta, previous caesarean section, maternal age over 35 years, preterm labour, induced labour, multiparity, previous uterine injury or surgery, uterine malformations, infection, and preeclampsia [1, 3, 6–8, 14–18]. It is believed that placenta accreta is becoming more common due to the rising caesarean section rate and advancing maternal age, both independent risk factors for placenta accreta [2, 17].

History of caesarean section and placenta previa are often of special interest as risk factors for placenta accreta. In a prospective observational cohort study of over 30,000 women who had caesarean delivery without labour, placenta accreta was present in 0.24% of women undergoing their first up to 6.74% of women undergoing their sixth or more caesarean delivery. In women with placenta previa the risk for placenta accreta was 3%, 11%, 40%, 61%, and 67% for first, second, third, fourth, fifth, and sixth or more caesarean deliveries. With every additional caesarean delivery the risk for emergency hysterectomy was rising as well. Hysterectomy was required in 0.65% for their first caesarean delivery and increased up to 8.99% for their sixth or more caesarean delivery [19].

In another study the incidence of placenta accreta in case of placenta previa was 5%. With a previous caesarean section, the incidence increased to 10% [1].

## 5. Avoiding Increased Blood Loss

Some studies showed promising results by injecting oxytocin into the umbilical cord, as it increased the rate of spontaneous expulsions of the placenta and fewer manual removals of the placenta, but two Cochrane reviews, either investigating umbilical cord injection of saline or oxytocin in the routine management of the third stage of labour [20] or for the reduction of MROP [21], were not able to detect a significant reduction in the need for MROP. Nevertheless, umbilical vein injection of oxytocin solution is an inexpensive and simple intervention that could be performed while placental delivery is awaited. However, high-quality randomized trials show that the use of oxytocin has little or no effect. The same review showed a statistically lower incidence in manual removal of placenta if prostaglandin solution was used. Unfortunately, there were only two small trials contributing to this meta-analysis [21].

Eller et al. published a study including 57 cases with placenta accreta, where all women underwent hysterectomy. In 15 cases an attempt was made to remove the placenta manually, but these entire women required immediate hysterectomy for uncontrollable bleeding. The authors of this study concluded that, in case of suspected placenta accreta, scheduled caesarean hysterectomy without attempting placental removal is associated with a significantly reduced rate of early morbidity compared with cases in which placental removal is attempted [22].

## 6. Diagnosis

Diagnosis of placenta accreta is not based on universally valid standard criteria but rather a diagnosis based on the obstetricians' impression and subjective judgement. Some authors use only clinical criteria for the diagnosis of placenta accreta, while others use histopathological criteria, which is not always possible for obvious reasons. Some authors distinguish between total and partial placenta accreta, a diagnosis even more difficult to make. As well for the term* placenta adherens* there is no consensus regarding exact criteria for the definition. This may also be contributing to the highly variable incidence of placenta accreta, with rates reported in literature between 1 : 93,000 and 1 : 110 [16].

Aside from patients with placenta previa and patients with a high risk of morbidly adherent placenta due to obstetrical history, the diagnosis of placenta accreta is usually made at the time of delivery. A prenatal screening for placenta accreta, especially for woman with risk factors, would be eligible. A prenatal diagnosis would allow a more planned approach and minimize maternal blood loss. In literature greyscale ultrasonography, colour Doppler imaging, and magnetic resonance imaging (MRI) have been described as alleged successful approaches to diagnose placenta accreta antenatally [6, 15, 17, 18]. Esakoff and colleagues stated that ultrasound examination is a good diagnostic test for accreta in women with placenta previa and found this in consistency with most other studies in the literature [23]. A recent meta-analysis involving 3707 pregnancies showed a sensitivity of 90.72% and a specificity of 96.94% of ultrasound for the antenatal detection of invasive placentation [24]. There is a general consensus that sensitivity and specificity of ultrasound are superior to those of MRI (sensitivity 80–85%, specificity 65–100%) [25], but often both imaging techniques are used in conjunction in women at risk. This is particularly true when the placenta is posterior and in obese women. However, prenatal diagnosis of placenta accreta in absence of further abnormalities of placentation remains a challenge.

There are also few biochemical markers named which are thought to have a diagnostic potential, such as elevated levels of maternal serum creatinine kinase, alpha fetoprotein, and *β*-human chorionic gonadotropin [18]. Others promisingly described cell-free fetal DNA, placental mRNA, and DNA microarray as potential tools for the diagnosis of abnormalities of placental invasion [15, 26, 27].

But so far there exists no diagnostic tool ready to use in daily routine for prenatal diagnosis of placenta accreta. The sensitivity of theoretically possible test methods also depends on the degree and extent of the abnormal placental invasion. In our experience prenatal diagnosis is almost impossible in the low risk population, where often the parturient is seen in the maternity hospital only for childbirth. We only can assume that these patients most probably have not undergone prenatal ultrasound examination with the very question of morbidly adherent placenta.

Nevertheless, the use of colour Doppler sonography in the third stage of labour has been promisingly introduced by Krapp et al. [6, 28]. They examined the third stage of labour using greyscale and colour Doppler ultrasound. In cases with normal placental separation they found cessation of blood flow between placenta and myometrium immediately after birth. Suggestive of placenta accreta was persistent blood flow from the myometrium deep into the placenta demonstrated by colour Doppler ultrasound. According to the authors this method allows a quicker diagnosis of placenta accreta and maternal blood loss can be minimized by early manual removal. As an ultrasound machine should be easily available in a well-equipped delivery unit it is advisable to use ultrasound in the third stage of labour complicated by retention of the placenta. With a gain of experience in judging the separation of the placenta from the uterine muscle ultrasound imaging may develop into a useful tool in the management of pathologic third stage of labour.

## 7. Treatment

**Figure 1:**
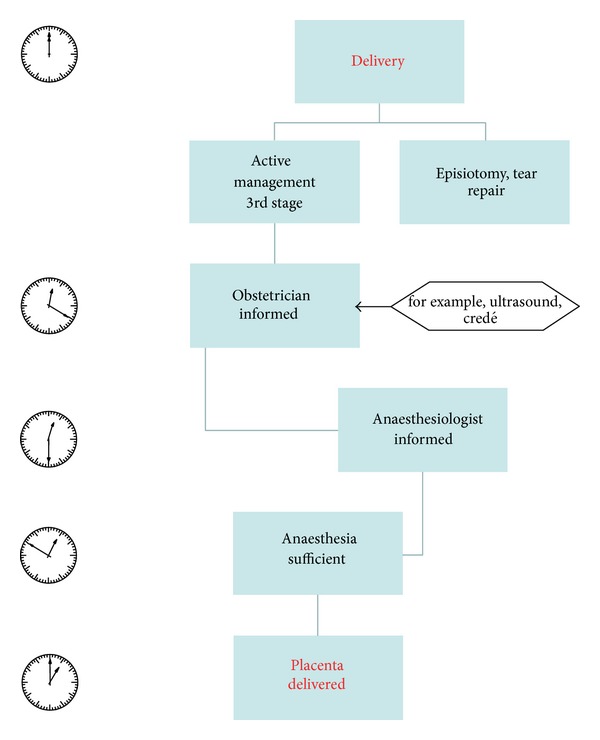
Flowchart for the treatment of retained placenta with special emphasis on the time frame.

Audureau et al. were able to show that the implementation of a multifaceted intervention scheme for the prevention and management of postpartum hemorrhage can be successful. In such way the median delay for second-line pharmacological treatment was significantly shortened from 80 min before introduction to 32.5 min afterwards [29]. Comparable to a strict work flow as already developed and implemented in most large delivery units for emergency caesarean section (target of decision delivery time < 20 min) a similar standardized protocol for manual removal of placenta might be useful. In Figure 1 we present a showcase flowchart for cases with retained placenta with special emphasis on the time frame. We believe that already strict observation of time, use of ultrasound for evaluation of the grade of placental detachment, and early involvement of support staff (i.e., second midwife, anaesthesiologist) might contribute to a reduction of maternal morbidity. Needless to say, the suggested time frame is only applicable in the absence of increased vaginal bleeding, and its efficacy has to be proven in a controlled trial. In case of an increased blood loss during third stage of labour ideally standardized operating procedures are already implemented.

In conclusion, retained placenta remains a problem of the third stage of labour, which in the low risk setting usually is occurring without prior warning. In daily routine adherence to a strict protocol of active management of third stage of labour may be helpful to minimize time interval between birth and delivery of placenta and therefore minimize postpartum complications. Further work is needed to proof this concept.

## References

[1] Mercier FJ, van de Velde M (2008). Major obstetric hemorrhage. *Anesthesiology Clinics*.

[2] Wise A, Clark V (2008). Strategies to manage major obstetric haemorrhage. *Current Opinion in Anaesthesiology*.

[3] ACOG Practice Bulletin (2006). Clinical Management Guidelines for Obstetrician-Gynecologists Number 76, October 2006: postpartum hemorrhage. *Obstetrics & Gynecology*.

[4] Cantwell R, Clutton-Brock T, Cooper G (2011). Saving mothers’ lives: reviewing maternal deaths to make motherhood safer: 2006–2008. The Eighth Report of the Confidential Enquiries into Maternal Deaths in the United Kingdom. *British Journal of Obstetrics and Gynaecology*.

[5] Weeks AD, Mirembe FM (2002). The retained placenta—new insights into an old problem. *European Journal of Obstetrics Gynecology and Reproductive Biology*.

[6] Krapp M, Baschat AA, Hankeln M, Gembruch U (2000). Gray scale and color Doppler sonography in the third stage of labor for early detection of failed placental separation. *Ultrasound in Obstetrics and Gynecology*.

[7] Weeks AD (2008). The retained placenta. *Best Practice & Research Clinical Obstetrics & Gynaecology*.

[8] Combs CA, Laros RK (1991). Prolonged third stage of labor: morbidity and risk factors. *Obstetrics & Gynecology*.

[9] Magann EF, Evans S, Chauhan SP, Lanneau G, Fisk AD, Morrison JC (2005). The length of the third stage of labor and the risk of postpartum hemorrhage. *Obstetrics & Gynecology*.

[10] Intrapartum Care. Care for healthy women and their babies during childbirth. http://www.nice.org.uk/nicemedia/live/11837/36280/36280.pdf.

[11] Ronsmans C, Graham WJ (2006). Maternal mortality: who, when, where, and why. *The Lancet*.

[12] Deneux-Tharaux C, Macfarlane A, Winter C (2009). Policies for manual removal of placenta at vaginal delivery: variations in timing within Europe. *British Journal of Obstetrics and Gynaecology*.

[13] Weeks AD, Alia G, Vernon G (2010). Umbilical vein oxytocin for the treatment of retained placenta (Release Study): a double-blind, randomised controlled trial. *The Lancet*.

[14] Khong TY (2008). The pathology of placenta accreta, a worldwide epidemic. *Journal of Clinical Pathology*.

[15] Mazouni C, Gorincour G, Juhan V, Bretelle F (2007). Placenta accreta: a review of current advances in prenatal diagnosis. *Placenta*.

[16] Bencaiova G, Burkhardt T, Beinder E (2007). Abnormal placental invasion: experience at 1 center. *Journal of Reproductive Medicine for the Obstetrician and Gynecologist*.

[17] Comstock CH (2005). Antenatal diagnosis of placenta accreta: a review. *Ultrasound in Obstetrics and Gynecology*.

[18] Rosen T (2008). Placenta accreta and cesarean scar pregnancy: overlooked costs of the rising cesarean section rate. *Clinics in Perinatology*.

[19] Silver RM, Landon MB, Rouse DJ (2006). Maternal morbidity associated with multiple repeat cesarean deliveries. *Obstetrics & Gynecology*.

[20] Mori R, Nardin JM, Yamamoto N, Carroli G (2012). Umbilical vein injection for the routine management of third stage of labour. *Cochrane Database of Systematic Reviews*.

[21] Nardin JM, Weeks A, Carroli G (2011). Umbilical vein injection for management of retained placenta. *Cochrane Database of Systematic Reviews*.

[22] Eller AG, Porter TT, Soisson P, Silver RM (2009). Optimal management strategies for placenta accreta. *British Journal of Obstetrics and Gynaecology*.

[23] Esakoff TF, Sparks TN, Kaimal AJ (2011). Diagnosis and morbidity of placenta accreta. *Ultrasound in Obstetrics and Gynecology*.

[24] D'Antonio F, Iacovella C, Bhide A (2013). Prenatal identification of invasive placentation using ultrasound: systematic review and meta-analysis. *Ultrasound in Obstetrics & Gynecology*.

[25] Berkley EM, Abuhamad AZ (2013). Prenatal diagnosis of placenta accreta: is sonography all we need?. *Journal of Ultrasound in Medicine*.

[26] Simonazzi G, Farina A, Curti A (2011). Higher circulating mRNA levels of placental specific genes in a patient with placenta accreta. *Prenatal Diagnosis*.

[27] Kawashima A, Sekizawa A, Ventura W, Koide K, Hori K, Okai T (2014). Increased levels of cell-free human placental lactogen mRNA at 28–32 gestational weeks in plasma of pregnant women with placenta previa and invasive placenta. *Reproductive Sciences*.

[28] Krapp M, Axt-Fliedner R, Berg C, Geipel A, Germer U, Gembruch U (2007). Clinical application of grey scale and colour Doppler sonography during abnormal third stage of labour: colour Doppler during abnormal third stage of labour. *Ultraschall in der Medizin*.

[29] Audureau E, Deneux-Tharaux C, Lefèvre P (2009). Practices for prevention, diagnosis and management of postpartum haemorrhage: impact of a regional multifaceted intervention. *British Journal of Obstetrics and Gynaecology*.

